# Heart Rate Variability Biofeedback Alleviates Subthreshold Depression by Reconstructing the Brain–Heart Axis via Habenular Network Functional Connectivity Modulation

**DOI:** 10.1002/cns.70692

**Published:** 2025-12-22

**Authors:** Xiaodan Xu, Yingnan Cao, Guiping Lin, Zhiyuan Long, Xiaojun Ouyang, Chang Liu, Zhoulei Li, Tinghuai Wang

**Affiliations:** ^1^ Sun Yat‐Sen University Guangzhou China; ^2^ Guangzhou XinHua University Guangzhou China

**Keywords:** autonomic nervous system, brain–heart axis, functional connectivity, habenula, heart rate variability, heart rate variability biofeedback, resting‐state functional MRI, subthreshold depression

## Abstract

**Aims:**

To investigate the role of the habenula (Hb)‐centered brain network in regulating the cardiac autonomic nervous system (ANS) in subthreshold depression (StD), and to explore the brain–heart axis mechanisms underlying the antidepressant effects of heart rate variability biofeedback (HRV‐BF) as a non‐pharmacological intervention.

**Methods:**

Thirty four StD participants and 32 healthy controls (HC) completed scale assessments (HAMD‐17, PHQ‐9, and PSQI) and cardiovascular measurements. StD participants received a 4‐week HRV‐BF with pre‐ and post‐rs‐BOLD fMRI. Bilateral Hb was used as the seed for ROI‐ and voxel‐wise functional connectivity (FC) analyses. To minimize potential confounding effects of sex imbalance, only female participants were included in voxel‐wise FC analysis (StD group, *n* = 8; HC group, *n* = 11) and pre‐post intervention comparisons in ROI‐wise FC analysis(StD group, *n* = 8).

**Results:**

StD participants exhibited elevated heart rate, reduced HRV indices (lnRMSSD, HF power, lnSDNN), and increased LF power. ROI‐wise FC analysis revealed that with increasing depression scores, z‐transformed functional connectivity (zFC) value between the Hb and the nucleus accumbens (NAc), ventral pallidum (VeP), amygdala, globus pallidus internus (GPi), and substantia nigra (SN) shifted from positive to negative, indicating a transition from functional connectivity coupling to anti‐coupling. Among these, Hb‐NAc and Hb‐VeP showed high discriminatory power (AUC = 0.876, *p* < 0.001). Voxel‐wise FC analysis demonstrated weakened Hb functional connectivity with several key regions within the default mode network, salience network, central executive network, visual network, and sensorimotor network, including precuneus, right posterior cingulate cortex, left middle cingulate cortex, right superior parietal lobule, cuneus, right calcarine cortex, left superior occipital gyrus, right paracentral lobule, and right postcentral gyrus. A 4‐week HRV‐BF intervention improved HRV indices and partially restored the functional connectivity of Hb‐centered networks, accompanied by significant improvements in depressive symptoms and sleep quality.

**Conclusion:**

Aberrant Hb‐centered functional connectivity may contribute to cardiac autonomic dysfunction in StD. Specifically, Hb‐NAc and Hb‐VeP functional connectivity may serve as neuroimaging biomarkers for StD. HRV‐BF enhances vagal tone, rebalances cardiac ANS function, and partly restores Hb‐centered brain network functional connectivity, thereby alleviating depressive symptoms and improving sleep quality. These findings highlight that Hb may be a key hub within the brain–heart axis and suggest this pathway as a promising early non‐pharmacological intervention target.

## Graphical abstract

Subthreshold depression (StD) participants exhibit cardiac autonomic dysfunction and aberrant functional connectivity (FC) of the habenula (Hb)‐centered network. Four‐week heart rate variability biofeedback (HRV‐BF), a non‐pharmacological intervention, has been shown to improve cardiac autonomic function and partially restore the functional connection of Hb‐centered networks, thereby alleviating depressive symptoms and enhancing sleep quality. AMY, amygdala; CEN, central executive network; DMN, default mode network; GPi, globus pallidus internus; NAc, nucleus accumbens; SMN, sensorimotor network; SN, substantia nigral; VeP, ventral pallidum; VN, visual network.

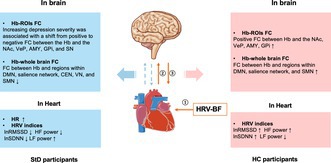

## 1 Introduction

Subthreshold depression (StD) refers to the presence of clinically significant depressive symptoms that do not meet the full diagnostic criteria for major depressive disorder (MDD), often accompanied by varying degrees of functional impairment or subjective distress. Epidemiological studies report StD prevalence rates ranging from 2.85% to 35.2% [1, 2], with higher incidence in females than males [2, 3]. University students, affected by academic pressure, interpersonal conflicts, and employment stress, represent a high‐risk population for StD [4, 5]. Previous research indicates that StD not only impairs quality of life, social functioning, and mental health but also significantly increases the risk of suicidal ideation and behaviors [1, 6], serving as a prodromal stage of MDD [7]. Therefore, early intervention for StD is critical to prevent progression to MDD.

Extensive evidence has demonstrated a significant association between MDD and cardiovascular diseases [8, 9]. The brain–heart axis, a bidirectional neural regulatory network linking the central nervous system (CNS) and the heart, has emerged as a key conceptual framework for understanding the comorbidity mechanisms of psychiatric and cardiovascular conditions [10, 11]. Patients with StD or MDD frequently exhibit cardiac autonomic nervous system (ANS) imbalance, characterized by increased sympathetic activity and decreased vagal tone [12, 13], reflecting impaired brain–heart axis regulation.

The habenula (Hb), particularly the lateral habenula (LHb), has recently emerged as a critical hub in the neurobiology of depression. The LHb encodes negative emotion and suppresses reward‐related signaling, contributing to depressive symptoms [14, 15]. Importantly, brain regions connected to the LHb via afferent and efferent pathways also interact with key nodes of the central autonomic network (CAN) [10, 16, 17], suggesting that the Hb may be involved in regulating the brain–heart axis, modulating cardiac autonomic function in StD.

Given the bidirectional nature of the brain–heart axis, peripheral modulation of autonomic activity may in turn influence central neural circuits through autonomic afferent pathways. Heart rate variability biofeedback (HRV‐BF), a non‐pharmacological neuromodulation technique that enhances vagal tone and restores autonomic balance [18, 19], provides an effective means to target this pathway. Through vagally mediated afferent signaling to emotion‐related brain regions, HRV‐BF has been shown to alleviate depressive symptoms via bottom‐up regulation of the CNS [19, 20]. However, whether its antidepressant effects involve modulation of Hb‐centered network connectivity remains unclear.

In this study, we aimed to investigate the role of the Hb‐centered brain network in regulating cardiac ANS activity in StD participants using resting‐state blood oxygen level‐dependent functional magnetic resonance imaging (rs‐BOLD fMRI) combined with cardiovascular measurements. Furthermore, we explored whether HRV‐BF exerted anti‐depressant effects by modulating Hb‐related brain network functional connectivity via vagal activation, thereby providing neurofunctional evidence for its application as an early, non‐pharmacological intervention for StD.

## 2 Methods

### 2.1 Participants

**FIGURE 1:**
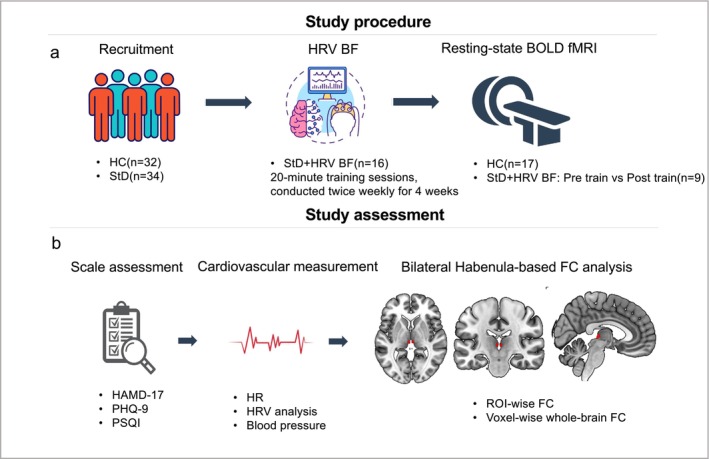
Workflow of the study. (a) Study procedure: Including participant recruitment, HRV‐BF training, and rs‐BOLD fMRI scanning. (b) Study assessments: Comprising scale assessment, cardiovascular measurement, and bilateral habenula‐based functional connectivity analysis. The red markers indicate the anatomical location of the bilateral habenula in axial, coronal, and sagittal planes. FC, functional connectivity; HAMD‐17, 17‐item Hamilton Depression Rating Scale; HR, heart rate; HRV, heart rate variability; PHQ‐9, Patient Health Questionnaire‐9; PSQI, pittsburgh sleep quality index.

A total of 34 university students with StD and 32 HC were recruited from Guangzhou Xinhua University. The two groups were matched for age and education level. Among the StD participants, 16 (15 females, 1 male) voluntarily underwent HRV‐BF training, of whom 9 (8 females, 1 male) completed resting‐state fMRI scans before and after the intervention. Additionally, 17 HC subjects completed one fMRI scan (Figure 1).

### 2.2 Ethics Statement

This study was conducted according to the Declaration of Helsinki. Ethical approval was obtained from the biological and medical ethics committee of Guangzhou Xinhua University. All participants gave informed consent.

### 2.3 Clinical Scale Assessments

Demographic data, including age, sex, and body mass index (BMI), were collected using standardized questionnaires. Depression severity was assessed by two psychiatrists or trained psychological counselors using the 17‐item Hamilton Depression Rating Scale (HAMD‐17) [21] and the Patient Health Questionnaire‐9 (PHQ‐9) [22]. Sleep quality was evaluated with the Pittsburgh Sleep Quality Index (PSQI) [23]. The scoring criteria for the scales are provided in the Supporting Information—Method Table S1.

### 2.4 Inclusion Criteria and Exclusion Criteria

#### 2.4.1 Inclusion Criteria

(1) StD Group: Participants aged 18–22 years, with HAMD‐17 scores between 8 and 17 [24] and/or PHQ‐9 scores between 5 and 14 [25]. They must meet 2–4 symptom criteria for MDD as defined by the Diagnostic and Statistical Manual of Mental Disorders, Fifth Edition (DSM‐5), including at least one core symptom (i.e., depressed mood or anhedonia), with symptoms persisting for more than 2 weeks; normal or corrected vision; no significant intracranial lesions on neuroimaging; no antidepressant usage within the last 4 weeks; right‐handed.

(2) HC Group: Participants aged 18–22 years, with HAMD‐17 scores < 8 and/or PHQ‐9 scores < 5; no history of psychiatric disorders; normal or corrected vision; no significant intracranial lesions on neuroimaging; right‐handed.

#### 2.4.2 Exclusion Criteria

Diagnosed with MDD, previous psychiatric or organic mental disorders; serious cardiovascular, cerebrovascular, hepatic, renal, hematological diseases; pregnant, lactating, or women planning pregnancy; secondary depression due to psychoactive substances or organic diseases; currently taking beta‐blockers, antidepressants, or other autonomic function‐affecting medications; history of alcohol or substance abuse; strong suicidal ideation; participation in other drug trials; antibiotic, probiotic, prebiotic, or synbiotic use within the past month; contraindications to MRI.

### 2.5 Heart Rate Recording and HRV Analysis

Pulse waves were recorded from the non‐dominant index finger using a photoplethysmographic (PPG) sensor. R–R intervals were extracted and analyzed for HRV using the BioTrace software integrated with the NeXus‐32 biofeedback system (Mind Media, the Netherlands).

#### 2.5.1 HRV Amplitude

HRV amplitude was calculated by detrending heart rate signals over two respiratory cycles and computing the root mean square of differences between consecutive positive and negative peaks, multiplied by 2 √2. Increased HRV amplitude reflects higher overall autonomic nerve regulation, influenced by both sympathetic and parasympathetic nervous systems.

#### 2.5.2 Time‐Domain Analysis [26]

Time‐domain metrics quantify variability in R–R intervals:

Standard deviation of normal‐to‐normal intervals (SDNN): Reflects overall autonomic nerve regulation.

Root mean square of successive differences (RMSSD): Mainly reflects vagally mediated HRV. Increased RMSSD indicates enhanced cardiac vagal modulation.

Both SDNN and RMSSD were natural log‐transformed (lnSDNN, lnRMSSD).

#### 2.5.3 Frequency‐Domain Analysis [26]

Power spectral density (PSD) was computed using fast Fourier transform (FFT), with frequency (Hz) on the x‐axis and PSD on the y‐axis to assess power distribution across frequency bands.

Low frequency (LF) power (0.04–0.15 Hz): Primarily reflects baroreflex‐mediated modulation of heart rate involving both sympathetic and parasympathetic inputs.

High frequency (HF) power (0.15–0.4 Hz): Reflects vagal modulation of heart rate; higher HF is associated with increased vagal tone.

Relative power (%) of LF and HF components was calculated as:

$$\mathrm{LF}\;\left(\%\right)=\frac{\mathrm{LF}\;\text{power}\left({\mathrm{ms}}^{2}\right)}{\text{Total power}\left({\mathrm{ms}}^{2}\right)}\times 100$$

$$\mathrm{HF}\;\left(\%\right)=\frac{\mathrm{HF}\;\text{power}\left({\mathrm{ms}}^{2}\right)}{\text{Total power}\left({\mathrm{ms}}^{2}\right)}\times 100$$

### 2.6 Blood Pressure Measurement

Blood pressure was measured in the brachial artery of the right upper arm using an automatic electronic sphygmomanometer (Omron HEM‐8102 K). Two consecutive readings were taken 1–2 min apart, and their average was used as the final blood pressure value. If the difference between the two systolic blood pressure (SBP) readings exceeded 10 mmHg or the difference in diastolic blood pressure (DBP) exceeded 5 mmHg, a third measurement was taken, and the average of the two closest readings was used.

### 2.7 HRV Biofeedback Training

The training protocol consisted of a pre‐training phase followed by formal training (details are provided in the Supporting Information—Method 1.2). Training was conducted in a quiet room with ambient temperature maintained between 24°C and 26°C. All participants were instructed to avoid consuming stimulants such as strong tea, coffee, or alcoholic beverages within 24 h prior to training.

### 2.8 Rs‐BOLD fMRI Data Acquisition

All participants underwent MRI scanning using a 3.0 T Siemens Prisma scanner (detailed parameters in Supporting Information—Method 1.3.1). During scanning, subjects wore noise‐reducing headphones, were instructed to avoid active thinking, and remained awake with eyes closed while lying still on the scanner bed.

### 2.9 MRI Data Processing and Analysis

#### 2.9.1 fMRI Preprocessing

Image data were processed using the RESTplus (v1.30) toolbox on MATLAB 2022a after a preprocessing step (Supporting Information—Method 1.3.2).

#### 2.9.2 ROI‐Wise Functional Connectivity Analysis Using Bilateral Habenula Seeds

Bilateral Hb regions were selected as seeds to perform ROI‐wise functional connectivity analysis with 13 predefined regions of interest (ROIs) reported to have afferent/efferent projections with the LHb [27], including bed nucleus of the stria terminalis (BNST), amygdala (AMY), nucleus accumbens (NAc), diagonal band nuclei (dBN), globus pallidus internus(GPi), ventral pallidum (VeP), substantia nigra(SN), lateral hypothalamic area(LHA), lateral geniculate nucleus(LGN), locus coeruleus(LC), ventral tegmental area (VTA), dorsal raphe nucleus (Raphe_D), and median raphe nucleus (Raphe_M). 13 ROIs were identified in established anatomical atlases. Partial Spearman correlation analyses were conducted to assess the associations between the Fisher z‐transformed functional connectivity (zFC) values of bilateral Hb‐each ROI and depressive scores (HAMD‐17/PHQ‐9 scores), while controlling for age and sex. Detailed ROI analysis procedures are described in the Supporting Information—Method 1.3.3.

#### 2.9.3 Voxel‐Wise Whole‐Brain Functional Connectivity Analysis Using Bilateral Habenula Seeds

Voxel‐wise functional connectivity maps were generated using bilateral Hb seeds. Between‐group comparisons were conducted in RESTplus (v1.30) with statistical thresholds set at voxel‐level *p* < 0.001 (uncorrected) and cluster‐level *p* < 0.05 (FWE‐corrected). Given that only one male participant in the StD group completed both pre‐ and post‐intervention rs‐fMRI scans, only female participants were included in the analysis to avoid potential confounding effects due to sex imbalance. The final sample consisted of 8 females in the StD group and 11 females in the HC group. Detailed analysis steps are provided in the Supporting Information—Methods 1.3.4.

#### 2.9.4 Receiver Operating Characteristic (ROC) Curve Analysis

ROC curve analysis was performed to evaluate the discriminative ability of bilateral Hb‐ROI functional connectivity (zFC values) to differentiate StD participants from healthy controls (Supporting Information—Method 1.3.5).

### 2.10 Statistical Analysis

Statistical analyses were performed using SPSS version 29.0. Continuous variables conforming to normal distribution were expressed as mean ± standard deviation (mean ± SD) and compared using independent samples *t*‐tests. Non‐normally distributed variables were described as median (interquartile range) [*M*(*P*_*25*_, *P*_*75*_)] and compared with the Mann–Whitney *U* test. Paired samples were analyzed using paired *t*‐tests if normally distributed, or Wilcoxon signed‐rank tests if not. Categorical variables were compared with Pearson's chi‐square (*χ* ^*2*^) tests. All tests were two‐tailed, with *p* < 0.05 considered statistically significant. Resting‐state fMRI preprocessing and functional connectivity analyses were conducted using RESTplus (v1.30) (detailed in Supporting Information—Method 1.3.6).

## 3 Result

### 3.1 Demographic Characteristics

There were no significant differences between the StD and HC groups in age, sex, or BMI (Table 1). However, scores on clinic scales showed that StD participants had significantly higher scores on the HAMD‐17, PHQ‐9, and PSQI compared to HCs (*p* < 0.05).

**TABLE 1:** Demographic information of the study participants.

	HC (*n* = 32)	StD (*n* = 34)	*t/Z/χ* ^*2*^	*p*
Age	20.00 (19.00, 20.00)	19.50 (19.00, 20.00)	−0.249	0.803^c^
Sex			2.569	0.109^d^
Male	10/32	5/34		
Female	22/32	29/34		
BMI(kg/m^2^)	21.13 ± 3.06	19.86 ± 2.28	−1.916	0.060^b^
Grade			2.084	0.353^d^
Freshman year	8/32	8/34		
Sophomore year	15/32	21/34		
Junior year	9/32	5/34		
HAMD‐17 score	2.00 (0.00, 3.00)	13.00 (9.00, 16.25)	−7.002	0.000^c^
PHQ‐9 score	0.00 (0.00, 1.00)	8.50 (5.75, 13.00)	−6.274	0.000^c^
PSQI score^a^	10.82 ± 7.78	16.00 ± 7.63	−1.634	0.117^b^

*Note:* Data are shown as mean ± SD, *M* (*P*_*25*_, *P*_*75*_), unless otherwise indicated. *p*‐value < 0.05 was considered statistically significant.

Abbreviations: BMI, body mass index; HC, healthy controls; StD, subthreshold depression.

^a^ Incomplete data: Due to incomplete questionnaire responses, the HC group (*n* = 17) and StD group (*n* = 9) were included in the PSQI score analysis.

^b^ Independent‐samples *t*‐test.

^c^ Mann–Whitney *U* test.

^d^ Pearson's chi‐square test.

### 3.2 Cardiac Autonomic Dysfunction Characterized by Reduced Vagal Tone and Relative Sympathetic Predominance in StD

**FIGURE 2:**
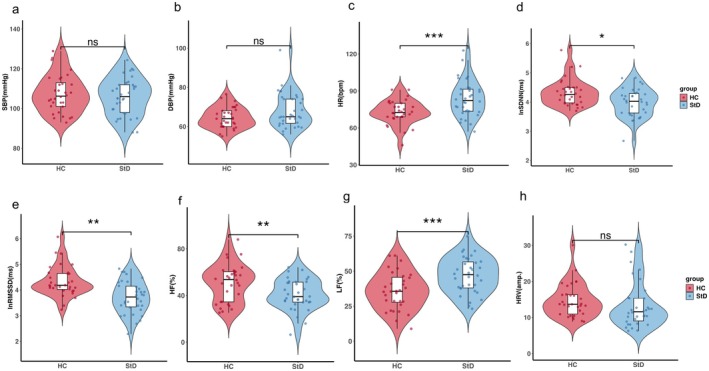
Group differences in HR, BP, and HRV parameters between individuals with StD and HC. (a–c) SBP, DBP, and HR; (d–e) lnSDNN and lnRMSSD; (f, g) HF power and LF power; (h) HRV amplitude. Variables including SBP, HR, HF power, and LF power followed a normal distribution and were analyzed using independent samples *t*‐tests. Non‐normally distributed variables were analyzed using the Mann–Whitney *U* test. Data are presented as violin plots with embedded boxplots (median and interquartile range) to illustrate the distribution of data and central tendency. * *p* < 0.05, **p < 0.01, ***p < 0.001. StD group (*n* = 34), HC group (*n* = 32). DBP, diastolic blood pressure; HF, high frequency; HR, heart rate; HRV amp., heart rate variability amplitude; LF, low frequency; lnRMSSD, natural logarithm of the root mean square of successive differences; lnSDNN, natural logarithm of the standard deviation of NN intervals; SBP, systolic blood pressure.

Compared to the HC group, participants in the StD group exhibited elevated HR (Figure 2c; *p* < 0.001), while no significant differences were observed in SBP or DBP (Figure 2a,b; *p* > 0.05).

HRV analysis revealed that the StD group had significantly reduced lnRMSSD, HF power (Figure 2e,f; *p* < 0.01), along with elevated LF power (Figure 2g; *p* < 0.001). The lnSDNN was significantly decreased (Figure 2d; *p* < 0.05), and HRV amplitude showed a decreasing trend (Figure 2h; *p* = 0.055), reflecting a potential reduction in overall autonomic regulation. These findings suggest that individuals with StD exhibit autonomic imbalance characterized by decreased vagal tone and relatively increased sympathetic activity compared to HCs (see Table S2) for full statistics.

### 3.3 Aberrant Functional Connectivity Between the Habenula and ROIs in StD

#### 3.3.1 Increasing Depression Severity Is Associated With a Shift Toward Negative Functional Connectivity Between the Habenula and Reward‐Affective‐Motor Related Brain Regions

**FIGURE 3:**
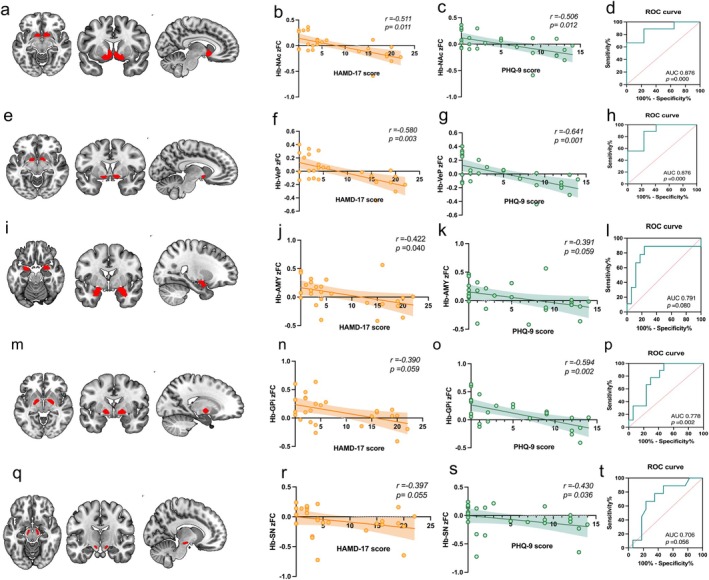
Habenula‐ROIs functional connectivity analysis (a, e, i, m, q) Axial, coronal, and sagittal views showing the anatomical locations of five ROIs: NAc, VeP, AMY, GPi, and SN. ROIs are marked in red. (b, f, j, n, r) Partial correlation analyses (Spearman) between Hb‐ROI zFC values and HAMD‐17 scores, controlling for sex and age. (c, g, k, o, s) Partial correlation analyses (Spearman) between Hb‐ROI zFC values and PHQ‐9 scores, controlling for sex and age. (d, h, l, p, t) ROC curves evaluating the discriminative ability of Hb‐ROI zFC values in distinguishing StD from HC. Hb‐NAc (AUC = 0.876, 95% CI, 0.699–1.000, *p* < 0.001), Hb‐VeP (AUC = 0.876, 95% CI, 0.725–0.980, *p* < 0.001), Hb‐AMY (AUC = 0.791, 95% CI, 0.556–0.974, *p* = 0.080), Hb‐GPi (AUC = 0.778, 95% CI, 0.582–0.941, *p* = 0.002) and Hb‐SN (AUC = 0.706, 95% CI, 0.484–0.892, *p* = 0.056). Total participants: 26 (StD group = 9, HC group = 17). AMY, amygdala; GPi, globus pallidus internus; NAc, nucleus accumbens; SN, substantia nigra; VeP, ventral pallidum.

**FIGURE 4:**
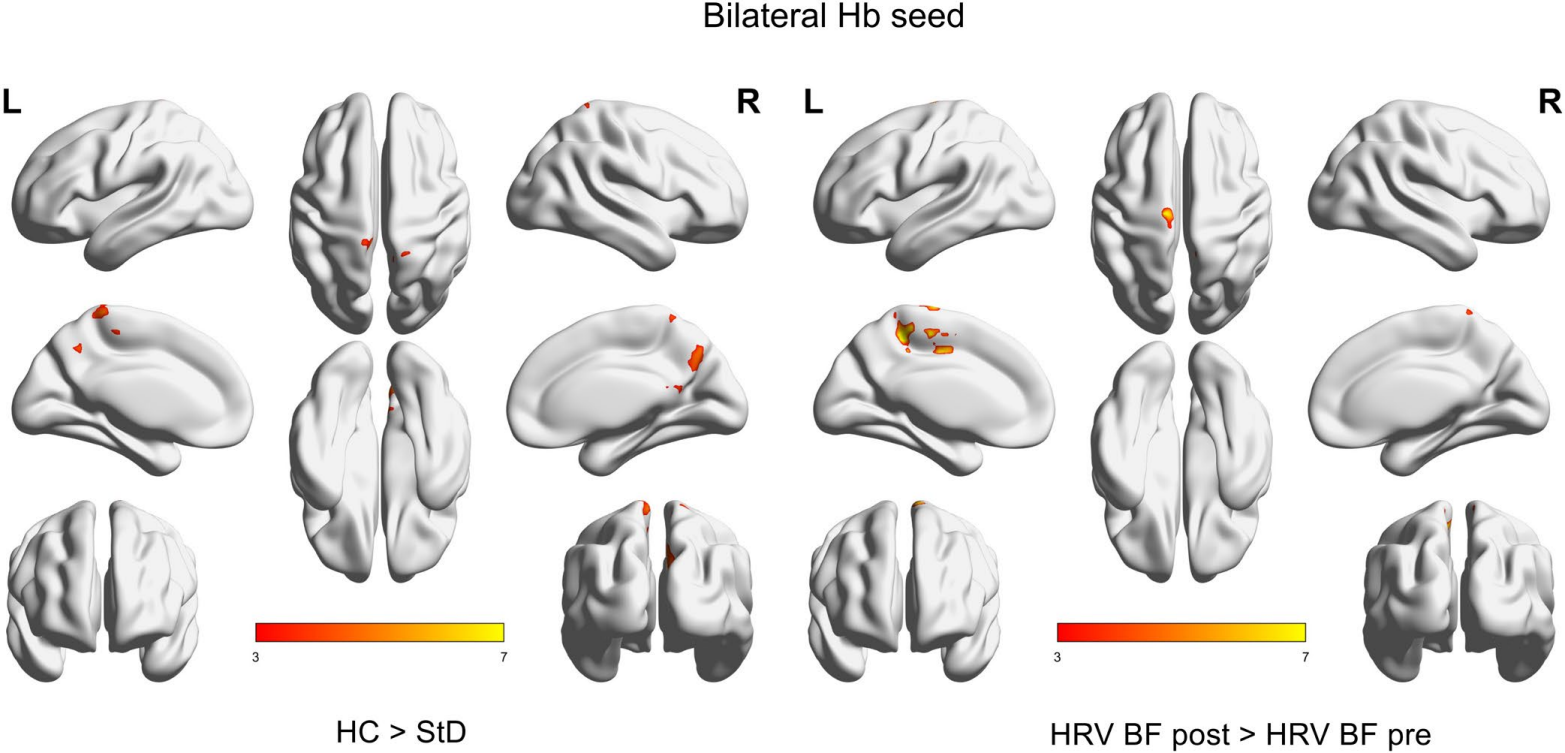
Whole‐brain voxel‐wise functional connectivity analysis based on bilateral habenula (Hb). Left panel: Brain regions showing significantly increased functional connectivity with bilateral Hb in healthy controls (HC) compared to individuals with subthreshold depression (StD) (HC > StD). Right panel: Brain regions showing significantly increased functional connectivity with bilateral Hb after HRV‐BF intervention compared to pre‐intervention (HRV‐BF post > HRV‐BF pre). Statistical threshold was set at voxel‐wise *p* < 0.001 (uncorrected), with a cluster‐level *p* < 0.05 (FWE corrected). Color intensity (from red to yellow) indicates the strength of functional connectivity with bilateral Hb. StD group (*n* = 8, female), HC group (*n* = 11, female).

Partial Spearman correlation analysis (controlling for age and sex) was performed to examine the relationship between bilateral Hb‐ROI functional connectivity and depression severity, as measured by HAMD‐17 and PHQ‐9 scores. As HAMD‐17 scores increased, the zFC values between the Hb and the NAc, VeP, AMY, GPi (*p* = 0.059), and SN (*p* = 0.055) progressively shifted from positive to negative, indicating a transition from functional coupling to anti‐coupling (Figure 3b,f,j,n,r). A similar pattern was observed with PHQ‐9 scores (Figure 4c,g,k,o,s), although the association with the AMY was only marginally significant (*p* = 0.059). No significant associations were observed for the remaining 8 ROIs (see Supporting Information Figure S1).

#### 3.3.2 ROC Curve Analysis Indicates Hb‐NAc and Hb‐VeP Connectivity as Potential Imaging Biomarkers for StD

To assess the discriminative power of Hb‐ROI functional connectivity (zFC values) in distinguishing StD participants from HC, receiver operating characteristic (ROC) curve analysis was conducted. Hb‐NAc and Hb‐VeP functional connectivity demonstrated strong discriminatory performance, each with an area under the curve (AUC) of 0.876 (Figure 3d,h; *p* < 0.001). Additionally, Hb‐GPi functional connectivity showed moderate classification performance (AUC = 0.778; Figure 3p; *p* < 0.05). The AUC values for Hb‐AMY and Hb‐SN functional connectivity were 0.791 (Figure 3l; *p* = 0.080) and 0.706 (Figure 3t; *p* = 0.056), respectively, and did not reach statistical significance. These findings indicate that altered functional connectivity between the Hb and NAc/VeP may serve as potential neuroimaging biomarkers for identifying individuals with stD.

### 3.4 Aberrant Habenula‐Centered Whole‐Brain Network Reorganization in StD

Voxel‐wise whole‐brain functional connectivity analysis using bilateral Hb as seed regions revealed that, compared to the HC group, the StD group exhibited significantly reduced functional connectivity between bilateral Hb and multiple brain regions, including precuneus and right posterior cingulate cortex (Default Mode Network, DMN); left middle cingulate cortex (Salience Network, SN); right superior parietal lobule(Central Executive Network, CEN); cuneus, right calcarine cortex, and left superior occipital gyrus(Visual Network, VN); right paracentral lobule and right postcentral gyrus(Somatomotor Network, SMN) (Figure 4, left panel; Table 2). These findings suggest that in individuals with StD, the habenula exhibits reduced functional connectivity with these regions may contribute to alterations in self‐referential processing, sensorimotor integration, and visuospatial attention.

**TABLE 2:** Whole‐brain voxel‐wise functional connectivity analysis based on bilateral habenula.

Condition	Voxels	Peak MNI coordinate (mm)			T value (peak intensity)	Peak MNI coordinate structure
		X	Y	Z		
HC > StD	226	3	−69	39	6.062	precuneus; cingulum_post_R; cuneus; calcarine_R; occipital_sup_L
	184	3	−51	69	6.577	cingulum_mid_L; paracentral_lobule_R; postcentral_R; parietal_sup_R
HRV BF post > HRV BF pre	79	−12	−39	51	12.820	precuneus; cingulum_mid_L; paracentral_lobule
	43	−9	−18	57	8.036	supp_motor_area_L; paracentral_lobule_L

*Note:* Statistical threshold was set at voxel‐wise *p* < 0.001 (uncorrected), with a cluster‐level *p* < 0.05 (FWE corrected). StD group (*n* = 8, female), HC group (*n* = 11, female).

Abbreviations: Calcarine_R, right calcarine cortex; cingulum_mid_L, Left middle cingulate cortex; cingulum_post_R, right posterior cingulate cortex; Peak MNI coordinate, primary peak locations in the Montreal Neurological Institute space; occipital_sup_L, left superior occipital gyrus; paracentral_lobule_L, left paracentral lobule; paracentral_lobule_R, right paracentral lobule; parietal_sup_R, right superior parietal lobule; postcentral_R, right postcentral gyrus; supp_motor_area_L, left supplementary motor area.

### 3.5 HRV‐BF Enhances Vagal Tone and Balances Autonomic Nervous Function

**FIGURE 5:**
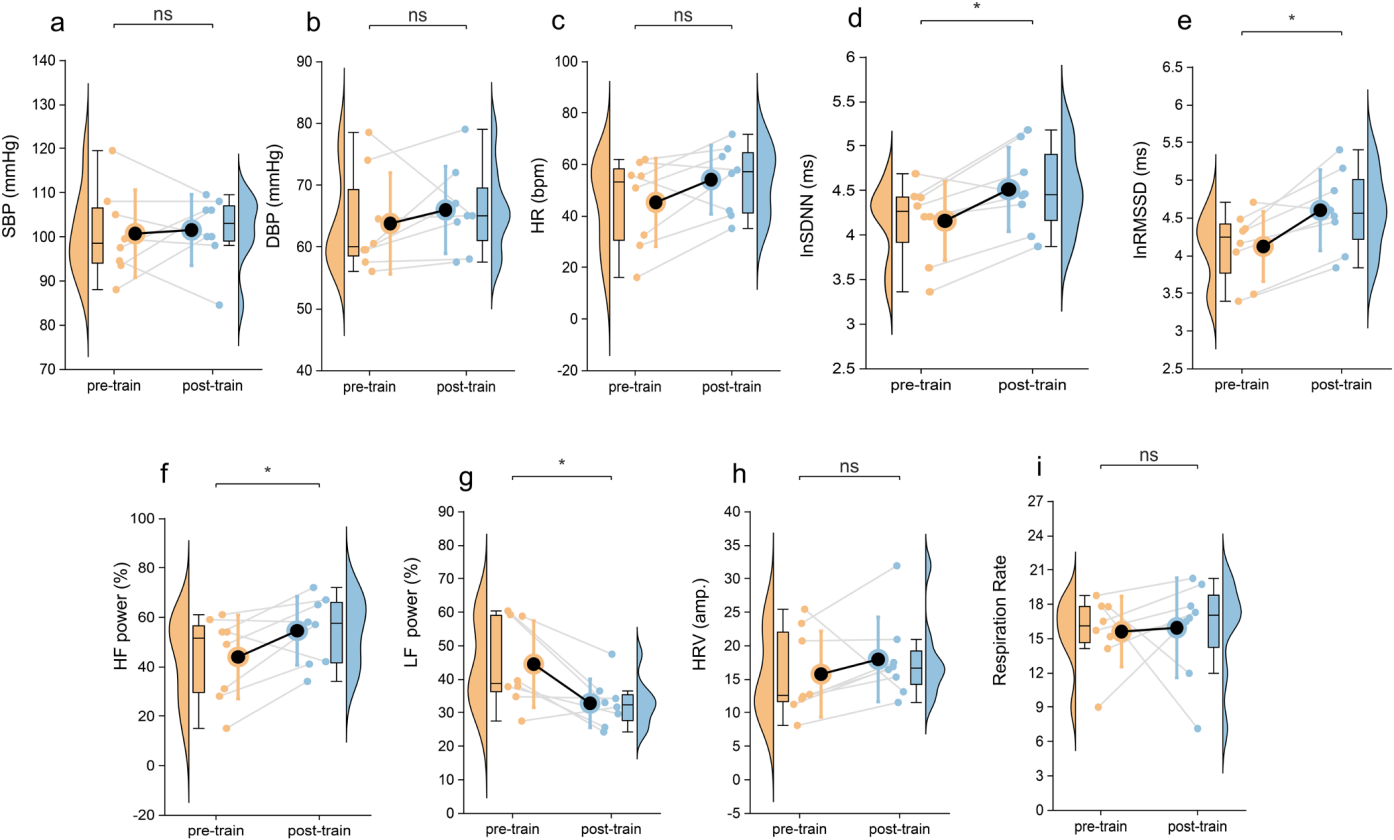
Effects of heart rate variability biofeedback on the autonomic nervous system. (a–c) Changes in cardiovascular physiological parameters: SBP (a), DBP (b), HR (c). (d, e) Changes in HRV time‐domain parameters: LnSDNN (d) and lnRMSSD (e). (f, g) Changes in HRV frequency‐domain parameters: HF power (f) and LF power (g). (h) Changes in HRV amplitude. (i) Changes in respiration rate. Paired‐samples *t*‐test was used to assess pre‐post differences, *n* = 8. * *p* < 0.05, **p < 0.01, ***p < 0.001.

After 4 weeks of HRV‐BF, participants with StD exhibited significant improvements in vagal‐mediated HRV parameters. Specifically, lnRMSSD and HF power significantly increased (Figure 5e,f; *p* < 0.05), along with lnSDNN (Figure 5d; *p* < 0.05). In contrast, LF power significantly decreased (Figure 5g; *p* < 0.05). No significant changes were observed in HRV amplitude (Figure 5h), SBP, DBP, HR (Figure 5a–c), or respiratory rate (Figure 5i). These results suggest that HRV‐BF increases vagal tone and restores autonomic balance.

### 3.6 Partial Restoration of Habenula‐Centered Brain Network Improves Depressive Symptoms and Sleep Quality Following HRV‐BF

**FIGURE 6:**
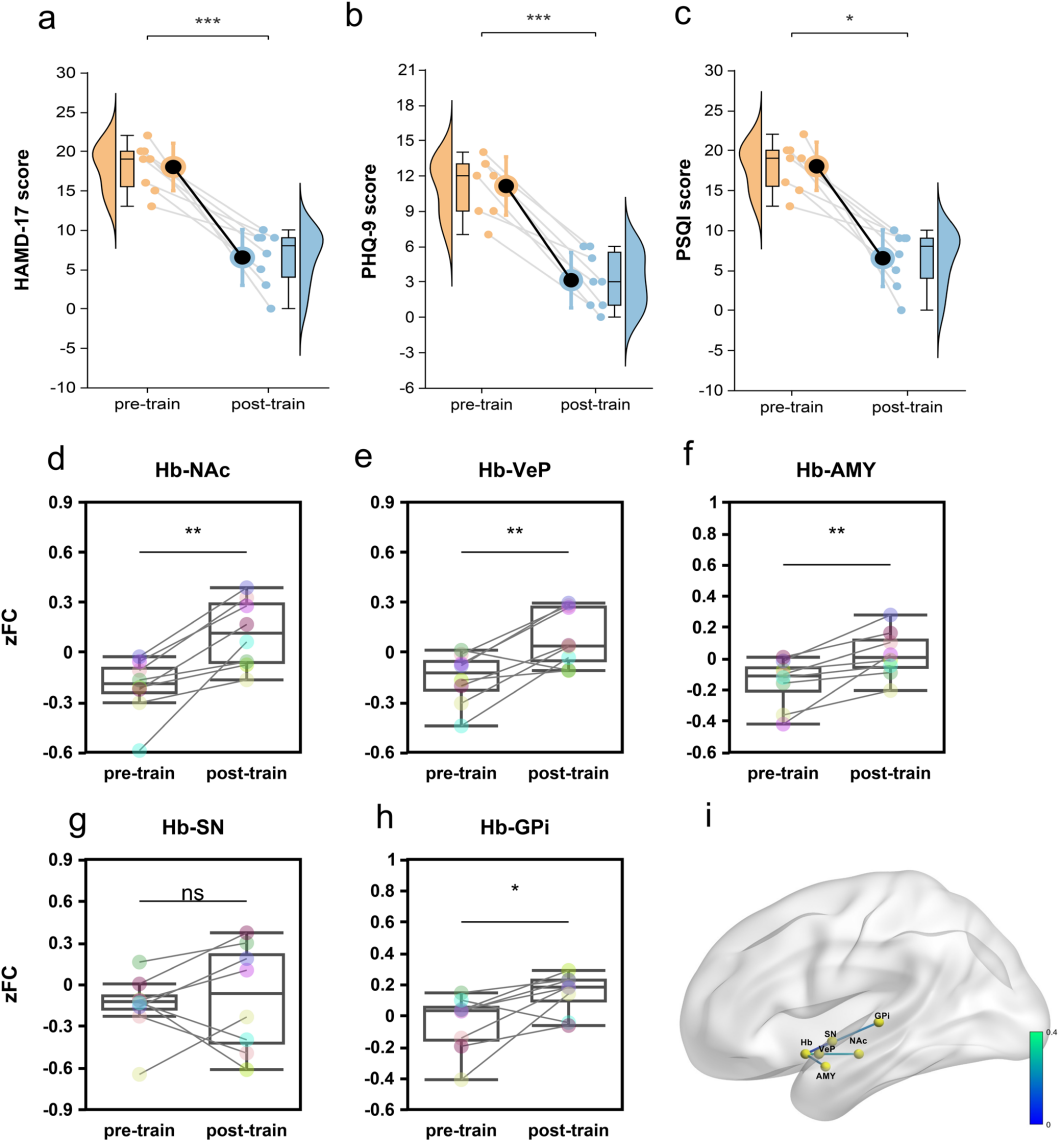
Effects of HRVBF on depressive symptoms, sleep quality, and bilateral habenular‐ROIs functional connectivity. (a–c) changes in scale assessments, including HAMD‐17 (a), PHQ‐9 (b), and PSQI (c). Paired raincloud plots summarize the intervention effect after HRVBF. Each plot integrates a half‐violin plot (showing kernel density estimation), a boxplot (indicating the median and interquartile range), individual paired data points with connecting lines (depicting within‐subject changes), and central mean ± SD bars (highlighting overall group‐level trends). *n* = 8. (d–h) show comparisons of zFC values between the bilateral Hb and ROIs before and after HRV‐BF training, including Hb‐NAc, Hb‐VeP, Hb‐AMY, Hb‐SN, and Hb‐GPi. Data are displayed as boxplots representing the median and interquartile range. *n* = 8. Normality of the pre‐post difference (delta values) was tested for each parameter (a–h). Variable dHAMD‐17 did not meet the normality assumption and was analyzed using the Wilcoxon signed‐rank test. The remaining variables were analyzed using paired‐samples *t*‐tests. * *p* < 0.05, ** *p* < 0.01, *** *p* < 0.001. (i) Functional connectivity changes (delta zFC value) of Hb with key regions (NAc, VeP, AMY, SN, and GPi) after HRV‐BF intervention. The color shift from blue to green indicates an increase in functional connectivity strength.

Post‐intervention results showed significant reductions in HAMD‐17, PHQ‐9, and PSQI scores (Figure 6a–c; *p* < 0.05), indicating substantial improvement in depressive symptoms and sleep quality. Neuroimaging data demonstrated significantly enhanced positive functional connectivity between the bilateral Hb and several reward‐affective‐motor‐related ROIs, including the Hb‐NAc (Figure 6d; *p* < 0.01), Hb‐VeP (Figure 6e; *p* < 0.01), Hb‐AMY (Figure 6f; *p* < 0.01), and Hb‐GPi (Figure 6h; *p* < 0.05). Among these, the upregulation of Hb‐NAc functional connectivity was the most robust (Figure 6i). Hb‐SN functional connectivity (Figure 6g) and Hb‐other ROIs showed no significant changes (see Figure S2). Furthermore, voxel‐wise analysis of whole‐brain Hb‐FC revealed post‐intervention increases in functional connectivity with regions including: precuneus (DMN); left middle cingulate cortex (Salience network); bilateral paracentral lobule and left supplementary motor area (SMN) (Figure 4), right panel; (Table 2). These findings suggest that HRV‐BF may alleviate depressive symptoms by modulating dysfunctional Hb‐centered brain network functional connectivity.

## 4 Discussion

Subthreshold depression (StD) is increasingly recognized as a prodromal stage of MDD. Individuals with StD already exhibit cardiac autonomic dysfunction, characterized by reduced vagal tone, relatively increased sympathetic activity, and decreased heart rate variability (HRV) [12], suggesting impaired central regulation of the brain–heart axis. The habenula (Hb), particularly the lateral habenula (LHb), is recognized as a critical hub for processing negative emotions and inhibiting reward, playing a key role in the pathogenesis of depression [15]. Moreover, brain regions connected to the LHb via afferent and efferent fibers have anatomical and functional links with central autonomic network (CAN) centers [10, 16, 17], suggesting that Hb may be involved in regulating the brain–heart axis, modulating cardiac autonomic function in StD.

Our integrated HRV and rs‐fMRI analyses revealed that StD participants exhibited concurrent cardiac autonomic imbalance and aberrant functional connectivity within the Hb‐centered network (Figures 2 and 3, Figure 4 left panel; Table 2), implying that the aberrant Hb‐centered network may weaken descending control of the brain–heart axis, thereby contributing to cardiac autonomic dysregulation.

Given the bidirectional nature of this axis, peripheral autonomic changes can also influence central networks via autonomic afferent pathways. Heart rate variability biofeedback (HRV‐BF), a non‐pharmacological neuromodulation technique that enhances vagal tone, has been shown to improve depressive symptoms by exerting “bottom‐up” regulation from the periphery to the brain. However, whether its antidepressant effects involve modulation of Hb‐centered networks remains unclear. In the present study, HRV‐BF increased vagal tone (Figure 5), partially restored Hb‐related network connectivity (Figure 4, right panel and Figure 6), and alleviated depressive symptoms in StD participants, supporting the role of the Hb as a regulatory node of the brain–heart axis and providing neurofunctional evidence for HRV‐BF intervention.

### 4.1 Impaired Central Regulation of the Brain–Heart Axis in StD

The brain–heart axis is a bidirectional regulatory network comprising the CAN, the intrinsic cardiac nervous system (ICNS), and the ANS [10]. CAN is the core component of the brain–heart axis, integrating information from cortical and subcortical regions, and dynamically regulating sympathetic and parasympathetic outflow via brainstem cardiovascular centers such as the rostral ventrolateral medulla (RVLM), nucleus tractus solitarius (NTS), dorsal motor nucleus of the vagus (DMV), and nucleus ambiguus (NA), etc. Through these descending pathways, the CAN modulates the ICNS to maintain cardiac autonomic homeostasis [10]. Chronic stress or depressive states can disrupt this regulation, resulting in cardiac autonomic dysregulation [28].

In this study, StD participants exhibited decreased vagal tone and increased sympathetic activity (Figure 2), indicating impaired descending regulation of the brain–heart axis. To investigate underlying central mechanisms, we examined Hb‐related functional connectivity using BOLD rs‐fMRI. Results showed aberrant Hb‐centered network connectivity in StD, specifically involving the NAc, VeP, AMY, SN and GPi (Figure 3). Voxel‐wise analysis revealed reduced functional connectivity between the Hb and the precuneus, right posterior cingulate cortex, left middle cingulate cortex, right superior parietal lobule, cuneus, right calcarine cortex, left superior occipital gyrus, right paracentral lobule, and right postcentral gyrus (Figure 4 and Table 2). Many of these regions are key nodes of large‐scale functional networks—including the default mode network (DMN), salience network (SN), central executive network (CEN), sensorimotor network (SMN), and visual network (VN). Reduced functional connectivity between Hb and these nodes may contribute to alterations in self‐referential processing, sensorimotor integration, and visuospatial attention, partially consistent with previous reports [29, 30]. Collectively, these findings indicate that disrupted Hb‐centered functional connectivity may contribute to brain–heart axis dysfunction in StD.

### 4.2 Potential Mechanisms of Hb‐Centered Brain Networks in Modulating Cardiac ANS

The LHb serves as a central hub for processing negative emotions and inhibiting rewards. To further elucidate how Hb‐centered network abnormalities influence cardiac autonomic regulation, we selected 13 brain regions anatomically connected to the LHb as regions of interest (ROIs) and examined their functional connectivity patterns [27]. Our results demonstrated that with increasing depressive severity, the functional connectivity between the Hb and several reward‐, affective‐, and motor‐related regions (NAc, VeP, AMY, SN, GPi) shifted from coupling to anti‐coupling (Figure 3). These regions maintain close structural and functional associations with the CAN [31, 32, 33, 34]. Functional anti‐coupling within these circuits may disrupt the integration between reward‐, affective‐, motor‐, and autonomic systems, thereby weakening adaptive regulation of the brain–heart axis.

Importantly, these five regions are also tightly linked to the midbrain monoaminergic system, including the ventral tegmental area (VTA) and the dorsal raphe nucleus (DRN) [34, 35, 36]. As an upstream regulator, the LHb inhibits dopaminergic (DA) neurons in the VTA and serotonergic (5‐HT) neurons in the DRN, thereby reducing monoaminergic output [27]. We therefore hypothesize that by suppressing these monoaminergic nuclei, the LHb reduces excitability in reward‐related, affective‐related, and motor‐related regions. Consequently, this cascade of events weakens functional integration with the CAN and ultimately impairs the central regulation of cardiac autonomic function.

Although this hypothesis was not directly tested in the present study, prior animal experiments provide strong supporting evidence: Electrical stimulation of the LHb increases mean arterial pressure (MAP) and decreases heart rate (HR), effects that are attenuated by DA and 5‐HT receptor antagonists or VTA lesions, suggesting that the LHb can modulate cardiovascular activity via the monoaminergic system [37, 38].

Notably, we did not directly observe functional connectivity abnormalities between Hb–VTA or Hb–DRN in the current study. However, previous research indicates that Hb–VTA functional connectivity anti‐coupling emerges as depression progresses to MDD [39], suggesting that functional disruption in the StD stage may still be at an early or compensatory stage, not yet manifesting as pronounced functional connectivity anti‐coupling. Moreover, ROC analysis showed that Hb–NAc and Hb–VeP functional connectivity effectively discriminated StD from HC (AUC = 0.876, *p* < 0.001), highlighting their potential as neuroimaging biomarkers with clinical relevance.

Collectively, these findings suggest that Hb‐centered network abnormalities in StD may impair integration between reward‐, affective‐, and motor‐regulating circuits and autonomic control centers, resulting in dysregulated brain–heart axis activity and cardiac autonomic imbalance.

### 4.3 Mechanisms of HRV‐BF in Remodeling the Brain–Heart Axis via Hb Networks

Our previous findings indicated that central regulation of the brain–heart axis is impaired in StD, manifested as Hb‐centered network abnormalities and reduced vagal tone. Given the bidirectional nature of the brain–heart axis, peripheral autonomic changes can influence central network plasticity and function through automatic afferent pathways, in addition to the descending control from the CNS [10]. Therefore, we hypothesized that enhancing vagal tone peripherally might restore impaired Hb‐centered network function and rebalance the brain–heart axis.

Heart rate variability biofeedback (HRV‐BF) provides a feasible approach to this hypothesis. As a non‐pharmacological neuromodulation technique, HRV‐BF enhances vagal activity through rhythmic breathing training at a resonance frequency (~0.1 Hz). Mechanistically, HRV‐BF increases vagal tone, enhancing signal transmission from the NTS to the CAN [19], thereby regulating sympathetic–parasympathetic balance. However, direct evidence demonstrating that HRV‐BF modulates Hb networks to remodel brain–heart axis integration has been lacking.

In the present study, after 4 weeks of HRV‐BF intervention, StD participants exhibited significantly increased vagal tone, with cardiac autonomic function shifting from sympathetic dominance toward parasympathetic predominance, indicating restored autonomic function (Figure 5). Concurrently, functional connectivity between the Hb and reward‐, affective‐, and motor‐related regions (NAc, VeP, AMY, GPi) was strengthened (Figure 6). Additionally, enhanced coupling was observed between the Hb and key nodes of large‐scale networks, including the DMN, Salience network, and SMN (Figure 4, right panel; Table 2). These results suggest that HRV‐BF may enhance Hb network function via “bottom‐up” vagal modulation. Restoration of Hb connectivity may in turn promote “top‐down” regulation of reward‐, affective‐, and motor‐related circuits with autonomic centers, forming a closed‐loop mechanism for brain–heart axis integration.

### 4.4 Limitations and Future Direction

This study has several limitations. First, the sample size was relatively small and female‐dominant (93.75%). While this reflects the higher prevalence of StD in women, it may limit the generalizability of the findings to male populations. Second, the 3.0 T MRI resolution precluded precise separation of LHb and MHb subregions, though selecting 13 ROIs anatomically connected to the LHb partially mitigated this limitation. Third, long‐term effects of HRV‐BF were not assessed, as the study focused on immediate changes after 4 weeks, though prior studies suggest sustained benefits [18, 40, 41]. Fourth, inter‐rater reliability was not formally calculated, but standardized training likely minimized scoring bias. Fifth, no active control group was included. A pre–post design in our study captured individual changes, and although placebo effects cannot be ruled out, the findings provide preliminary evidence supporting HRV‐BF's potential benefits. Future studies should recruit larger, gender‐balanced cohorts, incorporate active control interventions, include long‐term follow‐up, and utilize multimodal neuroimaging techniques (e.g., DTI, PET) to further elucidate the neural mechanisms and support clinical translation of HRV‐BF in subthreshold and major depression.

## 5 Conclusion

Aberrant functional connectivity within the Hb‐centered brain network may represent a potential central mechanism underlying cardiac autonomic dysfunction in StD. Hb‐NAc and Hb‐VeP functional connectivity may serve as neuroimaging biomarkers for early StD identification. HRV‐BF can enhance vagal tone, partially restore Hb network connectivity, and thereby remodel the brain–heart axis, contributing to antidepressant effects. These findings suggest that the Hb may serve as a critical central node of the brain–heart axis and highlight the axis as a potential early non‐pharmacological target for intervention.

## Author Contributions

**Xiaodan Xu:** participant recruitment, clinical assessments, HRV biofeedback training, data collection and analysis, drafting and revising the manuscript. **Yingnan Cao:** HRV biofeedback training. **Guiping Lin:** study design and data analysis. **Zhiyuan Long:** HRV biofeedback training. **Xiaojun Ouyang:** HRV biofeedback training. Chang Liu: participant recruitment, clinical assessments, HRV biofeedback training. **Zhoulei Li:** manuscript revision and funding acquisition. **Tinghuai Wang:** study design, manuscript revision, and funding acquisition. The authors have read and approved the final manuscript.

## Funding

This study was supported by the National Natural Science Foundation of China, 82271585, and the Natural Science Foundation of Guangdong Province, 2023A1515010388.

## Ethics Statement

This study was approved by the Biological and Medical Ethics Committee of Guangzhou Xinhua University (Approval ID:2022X001). All participants provided written informed consent before participation, and all procedures complied with the Declaration of Helsinki.

## Conflicts of Interest

The authors declare no conflicts of interest.

## Supporting information


**Table S1:** Scoring criteria for depression and sleep quality scales.
**Table S2:** Group differences in HR, BP, and HRV parameters between individuals with StD and HC.
**Figure S1:** Habenula‐ROIs functional connectivity analysis (a, d, g, j, m, p, s, v) Axial, coronal, and sagittal views display the anatomical locations of ROIs: BNST, dBN, LGN, LHA, Raphe_D, Raphe_M, VTA, and LC, marked in red. (b, e, h, k, n, q, t, w) Partial correlation analyses (Spearman) between Hb–ROI zFC values and HAMD‐17 scores, controlling for sex and age. (c, f, i, l, o, r, u, x) Partial correlation analyses (Spearman) between Hb–ROI zFC values and PHQ‐9 scores, controlling for sex and age. Total participants: 26 (StD group = 9, HC group = 17). BNST, bed nucleus of the stria terminalis; dBN, diagonal band nuclei; LC, locus coeruleus; LGN, lateral geniculate nucleus; LHA, lateral hypothalamic area; Raphe_D, dorsal raphe nucleus; Raphe_M, ventral raphe nucleus; VTA, ventral tegmental area.
**Figure S2:** Effects of heart rate variability biofeedback (HRVBF) on habenular‐ROIs functional connectivity. (a–h) show comparisons of zFC values between the bilateral Hb and ROIs before and after HRV‐BF training, including Hb–BNST, Hb–dBN, Hb–LGN, Hb–LHA, Hb–Raphe_D, Hb–Raphe_M, Hb–VTA, Hb–LC. Data are displayed as boxplots representing the median and interquartile range. Normality of the pre–post difference (delta values) was tested for each parameter, and a paired‐samples t‐test was used to assess pre–post differences. *n* = 8. * *p* < 0.05, ** *p* < 0.01, *** *p* < 0.001.

## Data Availability

The data that support the findings of this study are available from the corresponding author upon reasonable request.
