# Supplementary material for: Heart Rate Variability Biofeedback Alleviates Subthreshold Depression by Reconstructing the Brain–Heart Axis via Habenular Network Functional Connectivity Modulation
Source: CNS Neurosci Ther. 2025 Dec 22;31(12):e70692. doi: 10.1002/cns.70692 (PMC12720014; doi:10.1002/cns.70692)
Supplement: Supplementary file 1 — Table S1: Scoring criteria for depression and sleep quality scales. Table S2: Group differences in HR, BP, and HRV parameters between individuals with StD and HC. Figure S1: Habenula‐ROIs functional connectivity analysis (a, d, g, j, m, p, s, v) Axial, coronal, and sagittal views display the anatomical locations of ROIs: BNST, dBN, LGN, LHA, Raphe_D, Raphe_M, VTA, and LC, marked in red. (b, e, h, k, n, q, t, w) Partial correlation analyses (Spearman) between Hb–ROI zFC values and HAMD‐17 scores, controlling for sex and age. (c, f, i, l, o, r, u, x) Partial correlation analyses (Spearman) between Hb–ROI zFC values and PHQ‐9 scores, controlling for sex and age. Total participants: 26 (StD group = 9, HC group = 17). BNST, bed nucleus of the stria terminalis; dBN, diagonal band nuclei; LC, locus coeruleus; LGN, lateral geniculate nucleus; LHA, lateral hypothalamic area; Raphe_D, dorsal raphe nucleus; Raphe_M, ventral raphe nucleus; VTA, ventral tegmental area. Figure S2: Effects of heart rate variability biofeedback (HRVBF) on habenular‐ROIs functional connectivity. (a–h) show comparisons of zFC values between the bilateral Hb and ROIs before and after HRV‐BF training, including Hb–BNST, Hb–dBN, Hb–LGN, Hb–LHA, Hb–Raphe_D, Hb–Raphe_M, Hb–VTA, Hb–LC. Data are displayed as boxplots representing the median and interquartile range. Normality of the pre–post difference (delta values) was tested for each parameter, and a paired‐samples t‐test was used to assess pre–post differences. n = 8. * p < 0.05, ** p < 0.01, *** p < 0.001. [file CNS-31-e70692-s001.docx]

**Supplementary material**

[1 Methods 1](#_Toc216331317)

[1.1 Scoring Criteria for Depression and Sleep Quality Scales 1](#_Toc216331318)

[1.2 Protocol of HRV Biofeedback 2](#_Toc216331319)

[1.3 MRI Data Acquisition and Analysis 4](#_Toc216331320)

[2 Results 7](#_Toc216331321)

[3 References 11](#_Toc216331322)

# 1 Methods

## 1.1 Scoring Criteria for Depression and Sleep Quality Scales

**Table S1 Scoring Criteria for Depression and Sleep Quality Scales**

| **Scale** | **Application** | **Score Range** | **Criteria for classification** |
| --- | --- | --- | --- |
| **HAMD-17**  (Hamilton Depression Rating Scale, 17-item) | Used to assess the severity of depression; commonly applied for monitoring treatment efficacy | 0 – 52 | 0–7: Normal  8–16: Mild depression  17–23: Moderately severe depression  ≥24: Severe depression |
| **PHQ-9**  (Patient Health Questionnaire-9) | Used for initial screening, monitoring, and epidemiological investigation of depression | 0 – 27 | 0–4: No depression  5–9: Mild depression  10–14: Moderate depression  15–19: Moderately severe depression  ≥20: Severe depression |
| **PSQI**  (Pittsburgh Sleep Quality Index) | Used to evaluate overall sleep quality | 0 – 21 | 0–5: Very good sleep quality  6–10: Good sleep quality  11–15: Fair sleep quality  16–21: Poor sleep quality |

## 1.2 Protocol of HRV Biofeedback

The HRV biofeedback (HRV BF) intervention consisted of two phases: a pre-training and a formal training session. All training sessions were conducted in a quiet room with a controlled ambient temperature maintained at 24–26°C. Participants were instructed to abstain from consuming stimulants such as strong tea, coffee, or alcoholic beverages within 24 hours before each training session.

1.2.1 Pre-training

Prior to the formal intervention, participants underwent a pre-training session designed to guide diaphragmatic breathing and determine each individual's resonant respiratory rate (RRR). Participants practiced diaphragmatic breathing at six preset breathing rates (7.5 bpm, 7 bpm, 6.5 bpm, 6 bpm, 5.5 bpm, and 5 bpm), with each rate maintained for 2 minutes and separated by a 1-minute rest interval. The RRR was calculated using the BioTrace software in conjunction with the NeXus-32 biofeedback system (Mind Media, Netherlands).

1.2.2 Formal Training

HRV BF group: Participants with StD underwent diaphragmatic breathing training based on their individual RRR. During sessions T1 to T4, participants performed breathing training guided by the RRR pacer, while observing their heart rate and respiratory curves in real time. The therapist set a target HRV amplitude for each individual. When the respiratory curve closely matched the heart rate curve, a state of cardiovascular resonance was achieved, resulting in maximal HRV amplitude, which was reinforced with auditory feedback (birdsong).

In sessions T5 to T8, participants were instructed to follow the heart rate curve directly: inhaling when the heart rate curve began to rise and exhaling as it declined. Birdsong feedback was provided when the respiratory and heart rate curves were well aligned. Through this process of real-time feedback and correction, participants learned to consciously regulate their breathing to optimize autonomic function.

The HRV BF intervention was conducted twice weekly for four weeks. Each session consisted of a 5-minute baseline, 20-minute biofeedback training, and a final 5-minute baseline, during which R-R intervals were recorded for HRV analysis. Blood pressure was measured before and after each session. Additionally, participants completed home-based breathing training twice daily for 10 minutes per session.

## 1.3 MRI Data Acquisition and Analysis

1.3.1 Rs-BOLD fMRI Data Acquisition

All participants underwent MRI scanning using a 3.0 T Siemens Prisma scanner at the Radiology Department of the First Affiliated Hospital of Sun Yat-sen University. During scanning, subjects wore noise-reducing headphones, were instructed to avoid active thinking, and remained awake with eyes closed while lying still on the scanner bed. BOLD imaging parameters were: TR = 2090 ms, TE = 30 ms, flip angle = 90°, matrix size = 74×74, slice spacing = 3.75 mm, slice thickness = 3 mm, 38 slices, FOV = 224×224 mm², scan time: 7 min 6 sec. T1-weighted MPRAGE structural imaging parameters were: TR = 2000 ms, TE = 2.32 ms, TI = 900 ms, flip angle = 8°, matrix size = 256×256, slice spacing = 0.9 mm, slice thickness = 0.9 mm, 192 slices, FOV = 256×240 mm², scan time: 4 min 8 sec.

1.3.2 fMRI Preprocessing

Resting-state fMRI data were preprocessed using RESTplus (v1.30) running on the MATLAB 2022a platform. The preprocessing pipeline included the following steps: (1) DICOM images were converted to NIFTI format; (2) the first 10 volumes were discarded to ensure magnetic equilibrium; (3) slice-timing correction was performed to adjust acquisition timing across slices; (4) head motion correction was applied to reduce motion artifacts; (5) spatial normalization was conducted by registering individual functional images to the Montreal Neurological Institute (MNI) space with a resampled voxel size of 3×3×3 mm³; (6) spatial smoothing was performed using a 6-mm full-width at half-maximum (FWHM) Gaussian kernel; (7) linear trends were removed (detrending); (8) nuisance signal regression was conducted to remove confounding signals from white matter, cerebrospinal fluid, and Friston’s 24 head motion parameters; and (9) temporal bandpass filtering (0.01–0.08 Hz) was applied.

Participants with head motion exceeding 2 mm or 2° of rotation in any direction were excluded. Ultimately, 9 patients in the StD group completed two rs-fMRI scans (pre- and post-intervention), and 17 healthy controls (HC) completed one rs-fMRI scan.

1.3.3 ROI-wise Functional Connectivity Analysis Using Bilateral Habenula as Seeds

Using the bilateral habenula (Hb) as seed regions, region-of-interest (ROI)-wise functional connectivity (FC) analysis was performed. The mean blood oxygen level-dependent (BOLD) time series for each ROI was extracted, and Pearson correlation coefficients were computed between bilateral Hb and each ROI to quantify FC strength.

A total of 13 ROIs were included, selected based on previously reported afferent/efferent projections with the LHb: bed nucleus of the stria terminalis (BNST), amygdala (AMY), nucleus accumbens (NAc), diagonal band nuclei (dBN), globus pallidus internus (GPi), ventral pallidum (VeP), substantia nigra (SN), lateral hypothalamic area (LHA), lateral geniculate nucleus (LGN), locus coeruleus (LC), ventral tegmental area (VTA), dorsal raphe nucleus (Raphe_D), and median raphe nucleus (Raphe_M).

All ROIs were defined using published atlases: the Human Subcortical Atlas^[1]^, the Human Hypothalamic Atlas^[2]^, and the Automated Anatomical Labeling atlas version 3 (AAL3) ^[3]^. ROI masks were extracted using RESTplus (v1.30) in MATLAB 2022a. Correlation coefficients were Fisher z-transformed to generate zFC values. Partial Spearman correlation analyses were conducted to examine the associations between the zFC values of the Hb-each ROI and depression scores (HAMD-17/PHQ-9 scores) , while controlling for age and sex.

1.3.4 Voxel-wise Whole-Brain Functional Connectivity Analysis Using Bilateral Habenula as Seeds

Voxel-wise functional connectivity analysis was conducted using the bilateral Hb as seed regions. Pearson correlation coefficients were computed between the seed time series and the time series of all voxels in the brain. Resulting FC values were transformed into z-values using Fisher’s r-to-z transformation to improve normality. Group-level comparisons were conducted using second-level analysis in RESTplus (v1.30). The statistical threshold was set at voxel-wise *p* < 0.001 (uncorrected) and cluster-level *p* < 0.05 (FWE-corrected). Significant clusters were visualized and anatomically localized using xjView (v9.6).

Given that only one male StD participant completed both pre- and post-intervention rs-fMRI scans, only female participants were included in the final analysis to avoid gender-related confounding. As a result, the final sample included 8 female StD participants and 11 female HCs.

1.3.5 Receiver Operating Characteristic (ROC) Curve Analysis

ROC curve analysis was performed to evaluate the discriminatory power of zFC values between the Hb and ROIs in distinguishing StD patients from healthy controls. Using group status (StD vs. HC) as the classification variable, the area under the curve (AUC) and 95% confidence intervals were computed for each Hb-ROI pair. The AUC and significance (*p*-value) were computed using the nonparametric approach in SPSS (version 29.0). To obtain more robust confidence intervals, 95% CIs of the AUC were further estimated using the bootstrap method (2,000 resamples) implemented in R Studio (version 2024.12.1+563). The AUC ranges from 0.5 to 1.0, with higher values indicating better discriminative ability.

1.3.6 Statistical Analysis of Functional Connectivity Analysis

Resting-state fMRI preprocessing and functional connectivity analyses were conducted using RESTplus (v1.30). For ROI-wise analyses, BOLD time series were extracted from bilateral Hb and 13 ROIs, with Pearson correlation coefficients computed and Fisher r-to-z transformed into zFC values. To further explore the relationship between Hb-ROI functional connectivity and depressive symptoms, Spearman partial correlation analyses were performed, controlling for age and sex. These analyses were conducted in R Studio (version 2024.12.1+563) using the ppcor package with the pcor.test() function to obtain partial correlation coefficients, *p*-values, and test statistics. Two-tailed tests with a significance level of *α = 0.05* were applied. Voxel-wise whole-brain functional connectivity analyses were also performed using RESTplus (v1.30). Multiple comparisons were corrected using family-wise error (FWE) correction with thresholds of voxel-level *p* < 0.001 (uncorrected) and cluster-level *p* < 0.05 (FWE-corrected).

# 2 Results

**2.1** **Cardiac Autonomic Dysfunction Characterized by Reduced Vagal Tone and Relative Sympathetic Predominance in StD**

Compared to the HC group, participants in the StD group exhibited elevated HR (*p* < 0.001), while no significant differences were observed in SBP or DBP (*p* > 0.05). HRV analysis revealed that the StD group had significantly reduced lnRMSSD, HF power (*p* < 0.01), along with elevated LF power (*p* < 0.001). The lnSDNN was significantly decreased (*p* < 0.05), and HRV amplitude showed a decreasing trend (*p* = 0.055), reflecting a potential reduction in overall autonomic regulation. These findings suggest that individuals with StD exhibit cardiac autonomic imbalance characterized by decreased vagal tone and relatively increased sympathetic activity compared to HCs.

**Table S2 Group differences in HR, BP, and HRV parameters between individuals with StD and HC**

|  | HC(n=32) | StD(n=34) | *t/Z/χ²* | *P* value |
| --- | --- | --- | --- | --- |
| Blood pressure (mmHg) |  |  |  |  |
| SBP | 107.45±9.40 | 105.91±9.49 | *-0.660* | *0.512^*^* |
| DBP | 64.00(60.00, 68.37) | 65.00(61.50, 74.25) | *-1.490* | *0.136*^&^ |
| HR (bpm) | 72.84±9.71 | 83.66±14.69 | *3.506* | *0.000^*^* |
| HRV amplitude (ms) | 13.65(10.89, 16.41) | 11.58(8.66, 15.37) | *-1.918* | *0.055*^&^ |
| LF power (%) | 35.99±13.42 | 47.13±12.73 | *3.459* | *0.000^*^* |
| HF power (%) | 50.80±15.99 | 40.91±13.13 | *-2.753* | *0.008^*^* |
| lnSDNN (ms) | 4.26(3.96, 4.50) | 4.07(3.61, 4.35) | *-2.066* | *0.039*^&^ |
| lnRMSSD (ms) | 4.18(4.00, 4.66) | 3.80(3.37, 4.30) | *-3.220* | *0.001*^&^ |

Variables, including SBP, HR, HF power, and LF power followed a normal distribution and were analyzed using independent samples t-tests. Non-normally distributed variables were analyzed using the Mann–Whitney U test. ^*^Independent-samples t-test; ^&^Mann-Whitney U test. *p*-value < 0.05 was considered statistically significant.

**2.2 Habenula-ROIs functional connectivity analysis**

Partial Spearman correlation analysis (controlling for age and sex) was performed to examine the associations between bilateral Hb-ROIs functional connectivity and depression severity, as measured by HAMD-17 and PHQ-9 scores, while no significant associations were observed for the 8 ROIs (BNST, dBN, LGN, LHA, Raphe_D, Raphe_M, VTA, and LC ).


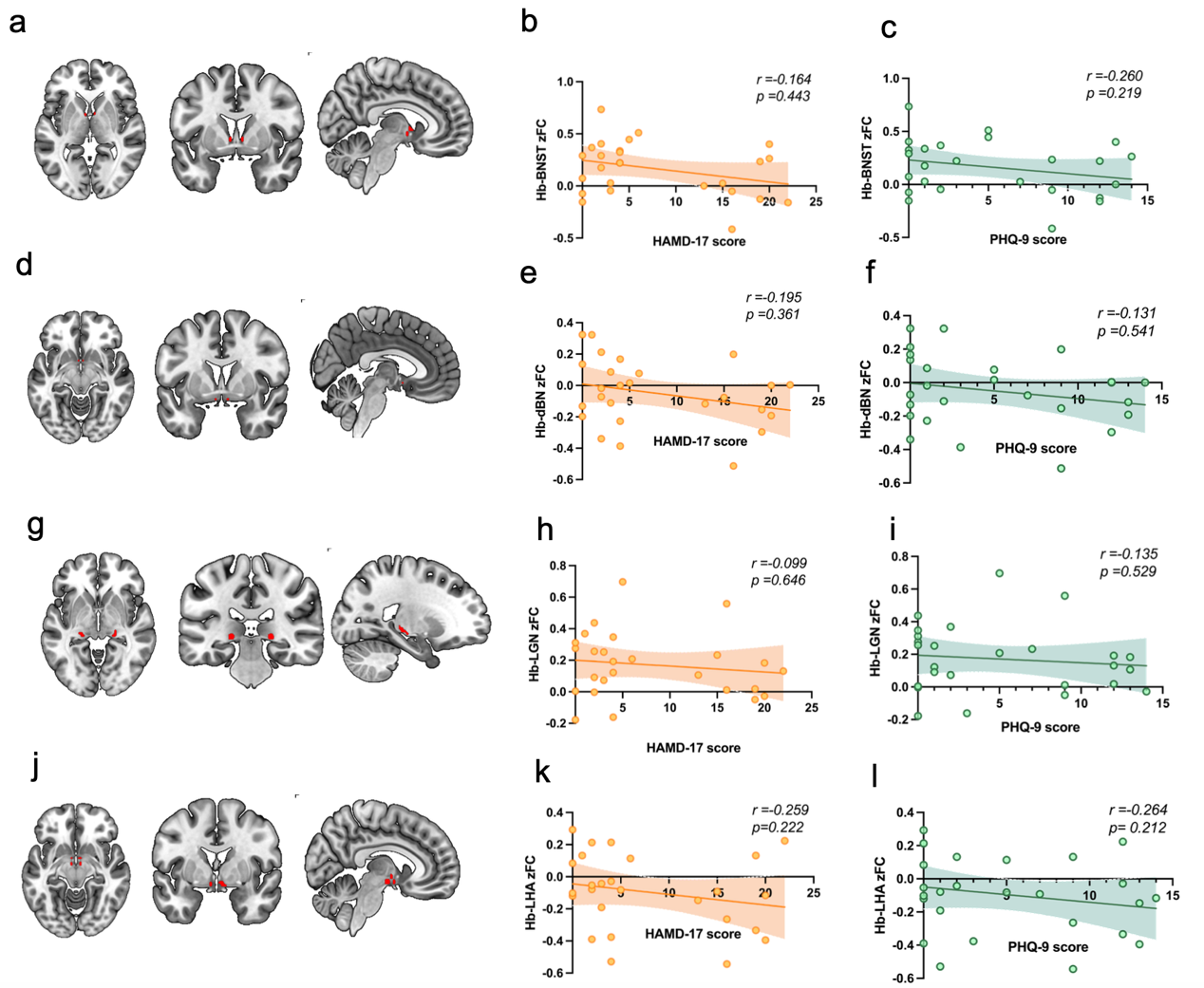


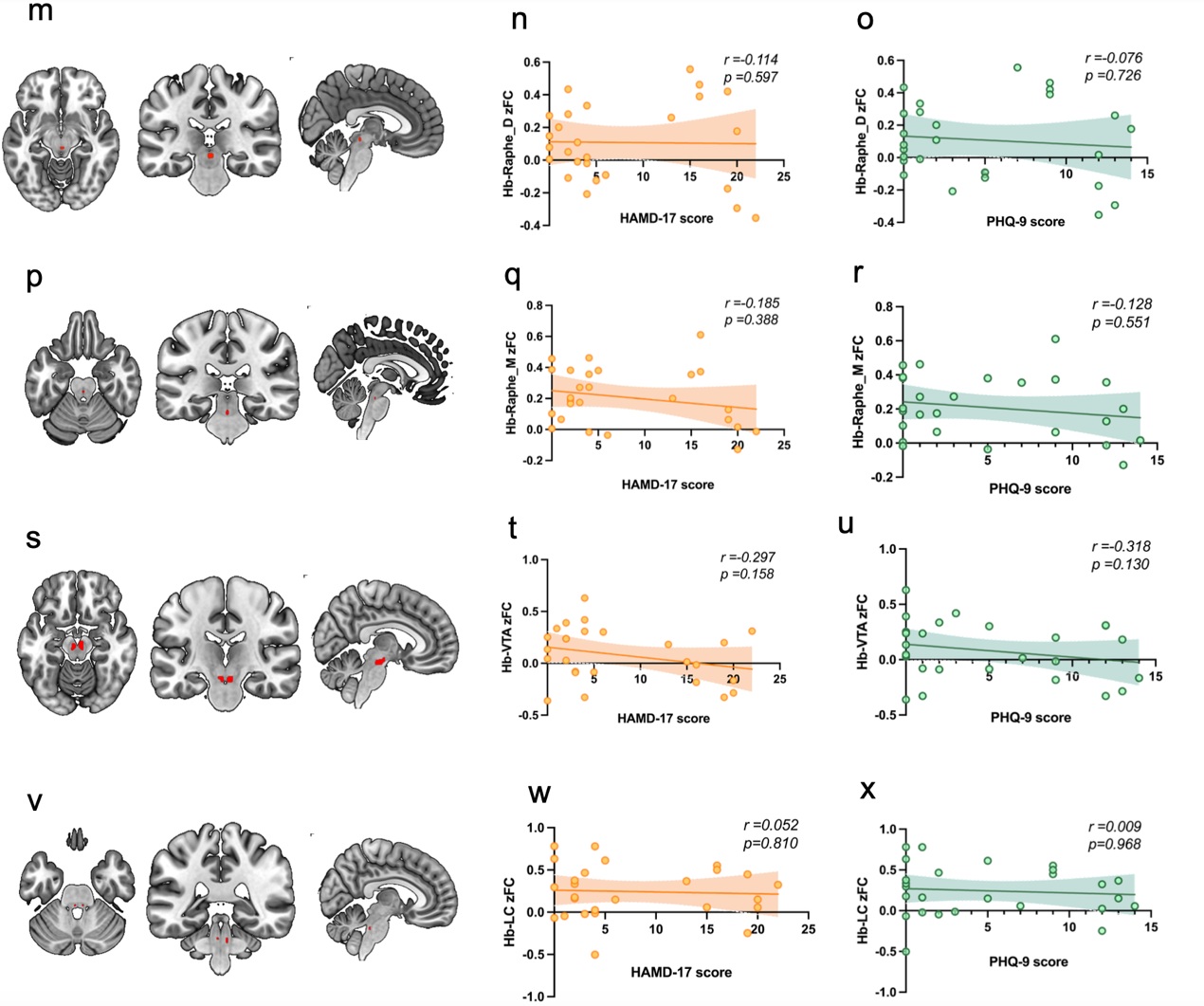


**Figure S1 Habenula-ROIs functional connectivity analysis** (a/d/g/j/m/p/s/v) Axial, coronal, and sagittal views display the anatomical locations of ROIs: BNST, dBN, LGN, LHA, Raphe_D, Raphe_M, VTA, and LC, marked in red. (b/e/h/k/n/q/t/w) Partial correlation analyses (Spearman) between Hb–ROI zFC values and HAMD-17 scores, controlling for sex and age. (c/f/i/l/o/r/u/x) Partial correlation analyses (Spearman) between Hb–ROI zFC values and PHQ-9 scores, controlling for sex and age. Total participants: 26 (StD group=9, HC group=17). Abbreviations: BNST, bed nucleus of the stria terminalis; dBN, diagonal band nuclei; LGN, lateral geniculate nucleus; LHA, lateral hypothalamic area; Raphe_D, dorsal raphe nucleus; Raphe_M, ventral raphe nucleus; VTA, ventral tegmental area; LC, locus coeruleus.

**2.3 Effects of heart rate variability biofeedback (HRV-BF) on habenular-ROIs functional connectivity**

After four weeks of HRV-BF, bilateral Hb-the remaining 8 ROIs showed no significant changes, including BNST, dBN, LGN, LHA, Raphe_D, Raphe_M, VTA, and LC.


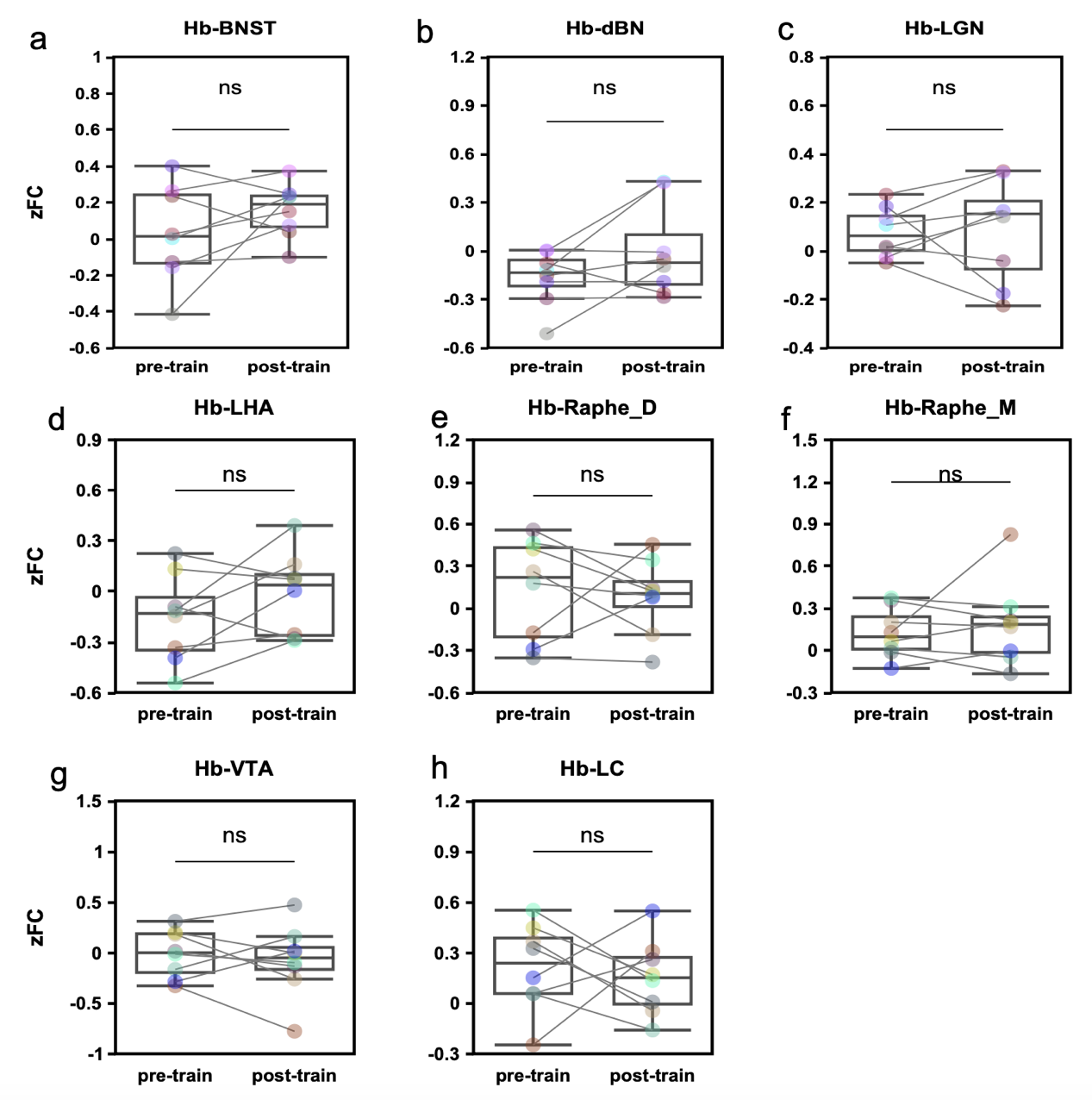


**Figure S2 Effects of heart rate variability biofeedback (HRVBF) on habenular-ROIs functional connectivity.** (a–h) show comparisons of zFC values between the bilateral Hb and ROIs before and after HRV-BF training, including Hb–BNST, Hb–dBN, Hb–LGN, Hb–LHA, Hb–Raphe_D, Hb–Raphe_M, Hb–VTA, Hb–LC. Data are displayed as boxplots representing the median and interquartile range. Normality of the pre–post difference (delta values) was tested for each parameter, and a paired-samples t-test was used to assess pre–post differences. n = 8. * *p* < 0.05, ** *p* < 0.01, *** *p*< 0.001.

# 3 References

1. Pauli WM, Nili AN, Tyszka JM. A high-resolution probabilistic in vivo atlas of human subcortical brain nuclei. Sci Data. 2018;5:180063. doi:10.1038/sdata.2018.63.

2. Neudorfer C, Germann J, Elias GJB, Gramer R, Boutet A, Lozano AM. A high-resolution in vivo magnetic resonance imaging atlas of the human hypothalamic region. Sci Data. 2020;7(1):305. doi:10.1038/s41597-020-00644-6.

3. Rolls ET, Huang CC, Lin CP, Feng J, Joliot M. Automated anatomical labelling atlas 3. Neuroimage. 2020;206:116189. doi:10.1016/j.neuroimage.2019.116189.
